# Price elasticity of demand for voluntary health insurance plans in Colombia

**DOI:** 10.1186/s12913-022-07899-2

**Published:** 2022-05-09

**Authors:** María Sofía Casabianca, Juan Miguel Gallego, Pamela Góngora, Paul Rodríguez-Lesmes

**Affiliations:** 1 School of Economics, Universidad del Rosario, Calle 12 C No. 4 – 69, 111711 Bogotá, Colombia; 2 Health Economics Research Centre, Nuffield Department of Population Health, University of Oxford, Oxford, UK

**Keywords:** Private health insurance, Voluntary private health insurance, Demand for health insurance, Price elasticity

## Abstract

**Background:**

Since 1993, Colombia has had a mandatory social health insurance scheme that aims to provide universal health coverage to all citizens. However, some contributory regime participants purchase voluntary private health insurance (VPHI) to access better quality health services (i. e., physicians and hospitals), shorter waiting times, and a more extensive providers’ network. This article aims to estimate the price elasticity of demand for the VPHI market in Colombia.

**Methods:**

We use data from the 2016–2017 consumer expenditure national survey and apply a Heckman selection model to address the selection problem into purchasing private insurance. Using the estimation results to further estimate the price semi-elasticity for VPHI, we then calculate the price elasticity for the households’ health expenditure and acquisition of VHPI.

**Results:**

Our main findings indicate that a 1% VPHI price increase reduces the proportion of households affiliated to a VPHI in the country by about 2.32% to 4.66%, with robust results across sample restrictions. There are relevant differences across age groups, with younger households’ heads being less responsive to VPHI price changes.

**Conclusions:**

We conclude that the VPHI demand in Colombia is noticeably elastic, and therefore tax policy changes can have a significant impact on public health insurance expenditures. The government should estimate the optimal VPHI purchase in order to reduce any welfare loss that the current arrangement might be generating.

**Supplementary Information:**

The online version contains supplementary material available at 10.1186/s12913-022-07899-2.

## Background

The literature in developed economies shows that private health insurance (PHI) is price inelastic [1, 2], with higher elasticity by age and income [3]. Moreover, it changes with the health system organisation, particularly in countries with universal health insurance, in which a PHI is complementary and voluntary to the health scheme plan. In these cases, elasticity tends to be higher but still inelastic or close to one. However, literature for developing countries is scarce, with no studies conducted in Latin American countries. Knowing the price elasticity of demand for PHI is crucial for better understanding the impact that fiscal measures may have on the demand for these services (i.e., changes in VAT or tax deductions consumers of PHI receive) [4]. Likewise, it is relevant information for companies that offer PHI to know how sensitive users are to price increases when planning premium adjustments each year.

Since 1993, Colombia has mandatory social health insurance scheme that aims to provide universal health coverage to all citizens. All affiliates of the Colombian Social Security Health System (known in Spanish as Sistema General de Seguridad Social en Salud – SGSSS) are entitled to a comprehensive health benefits package that covers nearly 98% of the Colombian population [5]. The health insurance scheme is financed through public resources and payroll contributions, which are mandatory for those able to pay. Formal workers and people with the capacity to pay are covered by the contributory regime, while those with low income and informal jobs can apply to a fully subsidised scheme [6]. In this system, health insurers, which receive a capitation-based premium per person, act as gatekeepers and limit healthcare access to contain health expenditures. In addition to the SGSSS, there is a voluntary private health insurance (VPHI) market for people who want to avoid the gatekeeping system, looking for faster access to health care, a more extensive pool choice of providers, and better amenities in health care facilities. In the country these products are also known as prepaid medicine, supplemental plans, or private health insurance plans. According to the article published by the Federation of Colombian Insurers – Fasecolda, in their magazine in 2015, about 4.4% of Colombian households have any type of VPHI given the data of the 2012 Longitudinal Social Protection Survey [7]. A more recent study conducted by PROESA about the VPHI in the Colombian healthcare system suggests that the VPHI market currently covers around 2.8 million people, which accounts for approximately 6% of the population [8]. VPHI are financed with private resources and usually offer supplementary coverage to the mandatory health plan (some offer complementary options as well). VPHI plans are in most cases paid by the household directly, and in some cases, employers might incentivise the acquisition of these plans. Public policy-makers have created a series of tax incentives for individuals who buy VPHI, hoping to relieve the burden on the public system [9, 10]. In Colombia, VPHI plans pay a reduced VAT rate (5% in contrast to the standard VAR rate of 19%), and VPHI payments are deductible up to a maximum of COP 581,000 monthly (USD $197 with the mean exchange rate of the survey year of 2.951 COP/USD). All VPHI plans are regulated by the Colombian law (Ley 1438 of 2011, Title IV) and companies that sell these plans are overseen by the National Superintendent of Health and the Financial Superintendence of Colombia [11]. Each VPHI plan and associated premiums are set in accordance with the technical guidelines defined by the National Superintendent of Health (Circular Externa No. 20 de 2015). Premiums are set based on individual’s age and gender, and they are not allowed to vary with health status. (see Additional file 2 for details about the VPHI market in Colombia).

This article aims to estimate the price semi-elasticity of demand for VPHI in Colombia, using the National Household Budget Survey (ENPH 2016–2017) [12]. The estimates are based on a model in which households choose whether to buy a VPHI or not. Household income, members’ age, the number of children under 18 years in the household, as well as the quality of health services that people can have access through the public health system are some of the main determinants of demand for VPHI and also of the premium paid by a given member of the household. The VPHI price or premium to be paid is an endogenous variable, making this the main methodological challenge when estimating price elasticity of demand for VPHI. To address this self-section into the VPHI system, we use a Heckman selection model. We instrument the regression of the logarithm of the risk premium (first stage) with the household size (number of household members), which we believe has an impact on the total value to be paid for the premium, but not the probability of purchasing insurance through another channel than the price. Our estimates suggest that the price elasticity at the household level ranges between -4.67 and -2.32, which has proven to be elastic and robust across the sample restrictions.

The rest of the paper is organised as follows. Following a brief literature review, "Methods" section describes the empirical strategy used to estimate de price elasticity of demand and the data. "Results" section presents the results and some robustness checks and heterogeneity exercise. "Discussion" section presents the discussion of the results and possible limitations of the estimation, and "Conclusions" section concludes.

### Literature review

The price elasticity of demand for VPHI refers to the degree of response of the quantity demanded of VPHI to changes in the premium or rate. More formally, the elasticity indicates the percentage change in the quantity demanded concerning a unit percentage change in the premium or the tariff, considering that the rest of the determinants of demand for VPHI, such as people’s income, remain constant [13]. There are two types of price elasticity of demand in this case: (i) elasticity of the decision to get insurance or not, also known as take-up elasticity, and (ii) elasticity of quantity or quantity level of coverage demanded, also called elasticity of demand. The vast majority of studies estimate the latter.

According to most of the estimates that the academic literature has documented, the demand for VPHI is price inelastic [1, 2]. Most studies are concentrated in the United States, where the estimated price elasticity ranges from -0.2 to -0.7, although a few studies find elasticities of -1 or even -2 [14]. Studies in other industrialised countries also suggest that this private health insurance demand is inelastic [15]. However, most of the literature highlights the possible heterogeneity results concerning the age of the individuals who decide whether to take insurance.

In countries with universal coverage schemes, and therefore, similar to the Colombian health system, price elasticity estimates differ between countries. In Spain, for example, a study in 2003 investigated the effect of the gap in quality of care between the National Health Service (NHS) and the services provided by private health insurance on the demand for health insurance volunteers (or private). Based on 400 surveys in Catalonia, the authors report a price elasticity of demand of -0.534. Although the magnitude is higher among individuals under 30 (-0.602), the demand is inelastic for the different age groups studied [3]. A recent study in Australia calculates the take-up elasticity and reports estimates in the range of − 0.32 to − 0.35 [16]. For the same country, some years prior, authors estimated various scenarios and found a price elasticity of demand (wide margin) that ranges between -0.29 and -0.52 [17]. This last estimate is similar to that reported in a previous study with data from a survey carried out in 2011 (from -0.4 to -0.6) [18]. For the Netherlands, the demand for private health insurance (of a supplementary type) is less responsive to price changes [19]. For example, in the population aged 35 to 44, the authors estimate a price elasticity of -0.17 among men and -0.13 among women. Like most studies, the youngest portion of the sample reports higher price elasticity magnitudes than the oldest. According to the systematic review conducted by Pendzialek, Simic, & Stock in 2014, in the case of duplicate or complementary private health insurance, in Canada, some authors report a price elasticity of demand of -0.46 [2].

Although income elasticity analysis is not the paper’s primary focus, related literature has shown that countries with similar health schemes as Colombia are around 1.22% and primarily positive. In a study in Spain, authors estimated the income semi-elasticity for a three-period panel data (2008–2014). They report that the cross-sectional models suggest that income effects increase between 2008 and 2014 (0.064 to 0.116, both at 1% significance level) [3]. However, when doing the panel estimation for 1928 Spanish households, results deceased and were non-significant at 5% [20]. For Catalonia, one of the most populated regions of Spain, authors report for the whole sample an income elasticity of 1.22 [3]. They also present estimations for age effects, showing that younger individuals have higher income elasticity, except when analysing individuals between 50 and 65 years old.

Despite all the studies that have been conducted in high-income economies, to the best of our knowledge, no study has estimated the price elasticity of demand for VPHI in low-middle income economies, including Colombia.

## Methods

### Empirical strategy

In the model we estimated, households choose whether to buy a VPHI or not. According to discrete choice theory, the household (or individual) decides to buy a VPHI when the expected utility of buying it is positive. This expected utility will depend on the insurance rate or premium, the characteristics of the service, the household’s epidemiological profile (measured by age, gender, and the demographic composition of the household), and household income. We model participation with the following probit model:

1 $${vphi}_{i}=\left\{\begin{array}{cc}1& \text{if } {\beta }_{0}+{\beta }_{1 }\cdot 1\mathrm{n}\left({p}_{i}\right)+{\beta }_{2}\cdot 1\mathrm{n}\left({y}_{i}\right)+{X}_{i}^{^{\prime}}\gamma +{\varepsilon }_{i}>0\\ 0& \mathrm{otherwise}\end{array}\right.$$

where the dependent (${vphi}_{i}$) is a dummy variable that equals 1 when the household reports expenses in VPHI and 0 otherwise. The first variable of interest is $p_{i}$, which is the monthly premium that households face to access the VPHI. The second variable of interest to this research is ${y}_{i}$, which is a proxy of household income. Also, there is a vector of observable characteristics ${X}_{i}$ as controls. For the head of the household, it includes gender dummies, age groups, and educational level. The household composition considers whether a person over 65 is present and the income level. There are also region-fixed effects. Finally, there is an error term ${\varepsilon }_{i} \sim N\left(\mathrm{0,1}\right)$ that includes all unobservable characteristics.

One of the leading and most important challenges faced in this estimation is that, in the data, we cannot observe the benchmark premium for households that decided not to buy VPHI. We approach this bias by correcting the selection using a Heckman selection model (or Heckit) as the one used by Cameron and Trived in their book Microeconomics: Methods and Applications [21]. This model consists of the following steps. First, we must estimate an auxiliary model to estimate the monthly premium households face to access the VPHI, conditional on a source of variation (an instrumental variable) that is not directly associated with the probability of buying VPHI apart from its effect through prices (exclusion restriction). The auxiliary model is estimated through a linear regression,

2 $$1\mathrm{n}\left({p}_{i}\right)={Z}_{i}^{^{\prime}}\delta +{u}_{i}$$

where the set of variables ${Z}_{i}$ includes ${X}_{i}$ and $1\mathrm{n}\left({y}_{i}\right)$ but additionally, it includes the number of members in the household, which acts as the instrument for the regression. The exclusion restriction, in this case, is that the household size affects the total value to be paid for the premium, but not the probability of buying insurance through any other channel than price. Additionally, it is assumed that ${u}_{i}\sim N\left(0,{\sigma }_{u}^{2}\right)$. The second step for the Heckit model to control for selection is to consider the correlation that exists by construction between participation in the VPHI and the value of the premium. For this purpose, consider the reduced form of Eq. 1, presented in Eq. 3.

3 $${vphi}_{i}=\left\{\begin{array}{cc}1& \text{if }{\eta }_{0}+{Z}_{i}^{^{\prime}}\iota +{\xi }_{i}>0\\ 0& \mathrm{otherwise}\end{array}\right.$$

The assumptions made over the possible distribution of unobservable variables in Eqs. 1 and 2 imply that $E\left({u}_{i}|{vphi}_{i}=1\right)=\rho \cdot \lambda$. Hence, $\rho$ is the correlation coefficient between unobservable variables in Eqs. 1 and 2, and $\lambda =\frac{\phi \left(-{\eta }_{0}-{Z}_{i}^{^{\prime}} \iota \right)}{1-\Phi \left(-{\eta }_{0}-{Z}_{i}^{^{\prime}} \iota \right)}$, known as the Mills inverse ratio, where $\phi \left(\bullet \right)$ is the density function of the normal function and $\Phi \left(\bullet \right)$ its cumulative function. Then, Eq. 3 is estimated, and $\lambda$ is calculated to be used as a regressor to estimate Eq. 2.

Finally, the structural participation model of Eq. 1 is estimated employing a two-stage least squares estimator, where the variable $1\mathrm{n}\left({p}_{i}\right)$ is instrumented with the predicted value of the auxiliary model of Eq. 2: $1\mathrm{n}\left({\widehat{p}}_{i}\right)={Z}_{i}^{^{\prime}} \widehat{\delta }$. This allows us to estimate the parameters of the unconditional model, despite the selection.

In order to compute both the price semi-elasticity of demand and the income semi-elasticity of demand, we need to derive the average marginal effects (AME) from Eq. 1. The price semi-elasticity corresponds to $AME\left({p}_{i}\right)=E\left[\frac{\partial \widehat{P}r\left({vphi}_{i}=1\right)}{\partial p/{p}_{i}}\right]$, and the income semi-elasticity to $AME\left({y}_{i}\right)$. With the semi-elasticities, we can compute the elasticities using the current proportion of VPHI users in Eq. 4:

4 $${\varepsilon }_{p}=AME\left({p}_{i}\right)\cdot \frac{1}{E\left({vphi}_{i}\right)},\text{ and }{ \varepsilon }_{y}= AME\left({y}_{i}\right)\cdot \frac{1}{E\left({vphi}_{i}\right)}$$

### Data

Data comes from the National Household Budget Survey, which we name ENPH due to the acronym in Spanish (Encuesta National de Presupuesto de los Hogares—ENPH 2016–2017). This survey is conducted every 4 years by the Colombian National Administrative Department of Statistics (Spanish: Departamento Administrativo Nacional de Estadística – DANE) [23].

To implement the model described, we used the urban sample of the ENPH 2016–2017 as the primary source of information, representing the best source for a detailed collection of how households decide to buy different goods and services and how much they spend on each other of them. The ENPH 2017 is representative at national level. The consumption of health services and VPHI are two of the goods and services analysed by this national-level survey. As this is a household expenditure survey, no specific data on the insurance policy characteristics are collected. Therefore, as presented in the model above, we only consider the VPHI. This survey also provides household-level information and the individuals who compose them that will be used for the proposed estimation model, particularly the instrument chosen to correct selection bias.

**Fig. 1:**
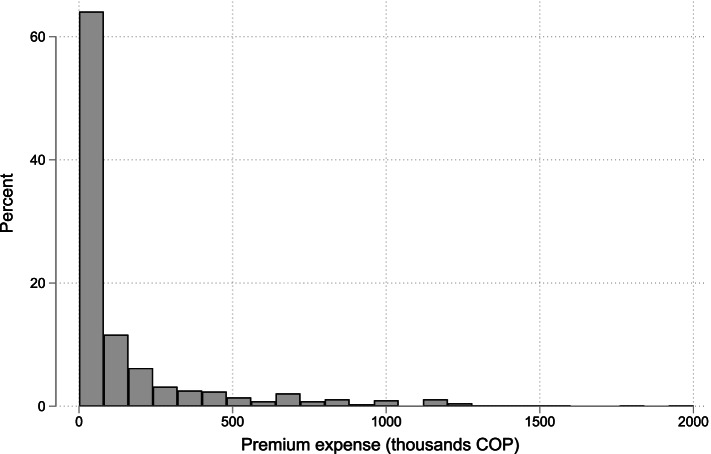
Distribution of the premium observed in the ENPH 2016–2017

The primary reference variable in this study is the insurance rate or monthly premium paid by households. This corresponds to expenditure item 12,530,101, “Annual payment for prepaid medicine or supplementary health plan”, which is specific for the premium^1^. Figure 1 presents this variable’s distribution for the national total of households that recorded expenditures in VPHI. As can be seen in this figure, there are minimum values for the premiums offered in the market; in fact, 65% of the observations are values of less than 30 USD (COP 90,000). This value corresponds to one of the cheapest monthly premiums for supplemental plans and prepaid medicine on the market^2^. A value below such figure is likely to relate to the fees of insurance policies linked to specific products, with significantly reduced coverage (ex. ambulance service, travel insurance) and premiums associated with other services which sometimes are compulsory products linked to other decisions (ex. school emergencies) [24]. As the data does not allow us to discriminate among them, we truncate the database on the prices that are likely to be associated with the monthly premium of a VPHI product. In our main results, we remove all premiums below 30 USD, and sensitivity analysis to this truncation cut-off is presented in the supplemental material for the paper for all specifications (see Table S2 and Figure A1 of additional file 1).

Table 1 shows the main characteristics of the households surveyed by the ENPH 2016–2017, which are consistent with what is expected of a household that decides to acquire a VPHI compared to households that do not have a registered expenditure on such items [25]. The table presents characteristics of VPHI users, defined both without and with the truncation. At first, it is observed that those households that report expenses in some additional or complementary health insurance scheme to the SGSSS (i.e., some payment for VPHI) have much higher income levels than those of the general population (6 or 9 million vs 1.2 million). Additionally, they are located mainly in the capital city – Bogotá—(30% to 40% vs 5%) and correspond to households with heads of household (HH) mainly older than 50 years (6% vs 48%). Furthermore, when analysing the labour market, almost all HH are in the formal sector of the economy (98% vs 62%) despite having the same employment rate. Finally, employees with a VPHI are more likely to work for the government or the private sector than self-employed or family workers. We will exploit all these characteristics of households and their members in the empirical exercise to have a fair estimate of the price elasticity for VPHI.

**Table 1:** Descriptive statistics according to imputed participation

	**No participants**		**VPHI user** **(no truncation)**		**VPHI user** **(Truncated below 30 USD)**	
	Mean	Std. Dev	Mean	Std. Dev	Mean	Std. Dev
Household Income (USD)	438.40	986.40	2,196.99	3,503.47	3,177.46	4,667.21
Premiums Expenditure (USD)			54.15	95.03	140.33	123.24
Male-Head of Household (HH)	0.58	0.49	0.62	0.49	0.65	0.48
HH age: Less than 30 y/o	0.12	0.33	0.04	0.21	0.03	0.18
HH age: 30 to 39 y/o	0.20	0.40	0.15	0.36	0.14	0.34
HH age: 40 to 49 y/o	0.21	0.41	0.18	0.38	0.20	0.40
HH age: 50 y/o or older	0.46	0.50	0.63	0.48	0.63	0.48
HH Education: Less than high school	0.50	0.50	0.08	0.27	0.03	0.17
HH Education: High school	0.28	0.45	0.07	0.25	0.04	0.18
HH Education: Tertiary education	0.22	0.41	0.85	0.35	0.94	0.25
At least one member over 65 y/o	0.26	0.55	0.49	0.75	0.54	0.79
Household income: Low	0.35	0.48	0.10	0.29	0.02	0.15
Household income: Middle	0.22	0.41	0.23	0.42	0.12	0.33
Household income: Middle-to-High income	0.08	0.26	0.66	0.47	0.85	0.36
Region: Atlántico	0.26	0.44	0.09	0.29	0.07	0.26
Region: Bogotá	0.05	0.21	0.30	0.46	0.40	0.49
Region: New departments	0.14	0.35	0.00	0.06	0.00	0.00
Region: Eastern	0.14	0.35	0.09	0.29	0.12	0.33
Region: Pacific	0.18	0.39	0.22	0.41	0.14	0.35
HH works	0.70	0.46	0.66	0.47	0.67	0.47
At least one member in the contributory regime	0.62	0.48	0.96	0.20	0.98	0.15
HH has an informal job	0.19	0.40	0.02	0.14	0.00	0.00
HH occupation: Private worker	0.37	0.48	0.49	0.50	0.47	0.50
HH occupation: Public worker	0.09	0.29	0.14	0.35	0.11	0.32
HH occupation: Employer	0.03	0.16	0.07	0.26	0.13	0.33
HH occupation: Other	0.52	0.50	0.29	0.46	0.29	0.45
Observations	80,659		626		213	

Only those who report expenses in expenditure item 12,530,101, “Annual payment for prepaid medicine or supplementary health plan,” is considered as participating households; the rest are assumed as unsubscribed to the service. In the case of the panel “Truncated below 30 USD”, only values above 30 USD/month are considered

## Results

In Table 2, we present the main results of the exercise. The first column presents results for the entire sample, and the second and third restrict the sample to households who are expected to be more willing to purchase the plans. Column 2 restricts the sample to non-informal workers, given that informal workers should not be able to access PVHI (affiliation to the mandatory health scheme is required first). For column 3, the value of the truncation cut-off that we chose was around 15% of the country’s monthly minimum wage at the time, showing that these premiums were an unaffordable option for low-income households. For this reason, there are around 40,000 observations in the first column (0.4% are VPHI users), 14,000 in the second (0.8% are VPHI users), and 8,000 in the last one (1.5% are VPHI users). Each value in the table corresponds to the estimator (average marginal effect) of the semi-elasticity value of the premium/income in each case. In general, it is found that a 1% more expensive premium reduces the proportion of households who decide to purchase a VPHI (price elasticity) by around 2 and 4 percentage points.

**Table 2:** Estimated price and income semi-elasticities and elasticities

Variable	(1)	(2)	(3)
	All	Non-informal	Middle-income or higher
Premium semi-elasticity, AME (${p}_{i}$)	-0.0194***	-0.0206***	-0.0346***
	(0.00352)	(0.00413)	(0.00715)
Income semi-elasticity, AME (${y}_{i}$)	0.0144***	0.0166***	0.0284***
	(0.00170)	(0.00170)	(0.00295)
Observations	41,153	14,378	8320
Proportion who spent on VPHI, E[${vphi}_{i}$]	0.00416	0.00862	0.0149
Calculated price elasticity (${\varepsilon }_{p}$)	-4.674	-2.389	-2.324
*p*-value	0.000	0.000	0.000
Calculated income elasticity (${\varepsilon }_{y}$)	3.459	1.921	1.905
*p*-value	0.000	0.000	0.000

Each column presents the marginal effects associated with the parameters ${\beta }_{1}$ and ${\beta }_{2}$ for Eq. 1, corresponding to the semi-elasticity concerning the premium and household income value. The estimation of the simultaneous equations model using data from the ENPH2017 includes (i) estimation of a Heckman selection model (household size is the instrument), (ii) estimation of ${\beta }_{1}$ and ${\beta }_{2}$ using the predictions of the premium considering counts the inverse of mills. VPHI users are defined as those who reported any expenditure above 30 USD in the expense article 12,530,101, “Annual payment of prepaid medicine or complementary health plan.” All models include controls for the head of the household, gender dummies, age groups, and educational level. From the household composition, it is considered whether a person over 65 is present and the income level. There are also region-fixed effects. Standard errors in parentheses. Significance: * 90%, ** 95%, ***99%

On the other hand, a 1% higher income is associated with a probability of between 1 and 3 additional percentage points of participation in the VPHI (income elasticity). Both price and income semi-elasticities are lower in the more restricted samples but still in the same order (magnitude). Given the observed levels of VPHI uptake, the computed price elasticity is around -4.67 to -2.32. If higher truncation cut-offs are selected, results are qualitatively the same, as shown in Table S2 of the supplemental material. This also shows that truncation plays a vital role in the estimation. Classifying all expenditures as valid premiums result in positive price semi-elasticities, which is inconsistent with the economic theory and the general literature^3^.

### Heterogeneity exercise

Table 3 presents the result of a heterogeneity exercise. Each value in the table corresponds to the estimator (average marginal effect) of the semi-elasticity value of the premium/income interacted with the variable related in the row in each case. Apart from the price and income semi-elasticities and elasticities, the table also presents the average expenditure (premium) reported for households. The proportion of households with VPHI according to the specific population can be found in the rows.

**Table 3:** Results of the heterogeneity exercise on semi-elasticities

	Mean Premium	Proportion who purchased a VPHI, E[${vphi}_{i}$]	Semi-elasticity (AME)	Computed elasticity
**Panel A**: Log premium spending				
HH Age:				
Less than 30 y/o (1)	255,483	0,00,279	-0.00165	-0.590
			(0.00247)	(0.888)
30 to 39 y/o (2)	286,093	0,00,844	-0.0293***	-3.471
			(0.00632)	(0.749)
40 to 49 y/o (3)	452,738	0,00,861	-0.0146**	-1.699
			(0.00540)	(0.627)
50 y/o or older (4)	464,654	0,00,969	-0.0231***	-2.382
			(0.00546)	(0.564)
At least one member over 65 y/o:				
No	394,215	0,00,809	-0.0197***	-2.430
			(0.00402)	(0.497)
Yes	516,750	0,0119	-0.0260***	-2.196
			(0.00623)	0.525
At least one child under 5 y/o:				
No	430,069	0,00,926	-0.0258***	-2.780
			(0.00493)	(0.532)
Yes	356,767	0,00,601	-0.0171***	-2.847
			(0.00444)	(0.738)
**Panel B**: Log household income				
HH Age:				
Less than 30 y/o (1)	255,483	0,00,279	0.00623**	2.234
			(0.00299)	(1.071)
30 to 39 y/o (2)	286,093	0,00,844	0.0178***	2.105
			(0.00286)	(0.339)
40 to 49 y/o (3)	452,738	0,00,861	0.0160***	1.858
			(0.00238)	(0.277)
50 y/o or older (4)	464,654	0,00,969	0.0186***	1.920
			(0.00235)	(0.242)
At least one member over 65 y/o:				
No	394,215	0,00,809	0.0158***	1.957
			(0.00168)	(0.208)
Yes	516,750	0,0119	0.0202***	1.707
			(0.00346)	(0.291)
At least one child under 5 y/o:				
No	430,069	0,00,926	0.0185***	1.998
			(0.00191)	(0.207)
Yes	356,767	0,00,601	0.0125***	2.083
			(0.00254)	(0.422)
Observations			14,378	

Each row presents the marginal effects associated with the parameters corresponding to the estimate shown in the columns name concerning the value of the premium and household income interacted with the age of the head of the household, if there is at least one member of the household over 65 years old [y/o] or if there are children under five y/o in the household. The estimation of the simultaneous equations model using data from the ENPH2017 includes (i) estimation of a Heckman selection model (household size is the instrument), (ii) estimation of ${\beta }_{1}$ and ${\beta }_{2}$ using the predictions of the premium considering counts the inverse of mills. The model followed is restricted to a minimum premium value of 30 USD and for the head of the household to work a non-informal job. All models include controls for the head of the household, gender dummies, age groups, and educational level. From the household composition, it is considered whether a person over 65 is present and the income level. There are also region-fixed effects present in the estimation. Standard errors in parentheses. Significance: * 90%, ** 95%, ***99%. The complete table with tests of the validity of the linear combination of parameters is in the supplemental material (see Table S4 in Additional file 1)

There is a differentiated effect of the premium to pay for the insurance according to the HH age. Households with heads under the age of 30 respond less to the price to pay for the VPHI than all other age ranges (the formal test of equivalence of the coefficients is presented in Table S4 in the Additional file 1). On the other hand, the probability of participation in the VPHI for HH in this same age range is higher given an income increase than it is for any other age range. We tested for differential effects among households with male HH and female HH and found no significant difference between the calculated elasticities for this exercise.

When there are household members over 65 y/o, households respond more to increases in the premium price than those without these members, yet the estimates are not statistically different. As income increases, households with members over 65 are less likely to participate in the VPHI market than their counterparts. On the other hand, children under five years of age in the home make the household more likely to react to price variations. However, once again, estimates are not statistically different from one another. When analysing the income elasticity estimates, we find that households with children under five y/o are more likely to acquire a VPHI plan when the household income increases.

## Discussion

The results of the first exercise, showing a price elasticity around -4.67 and -2.32, indicate a notoriously elastic demand for VPHI in Colombia. In addition, income elasticity is around 1.9 and 3.45, suggesting that a 1% higher income is associated with an increase in the demand of VPHI of around 1.9% to 3.45%. As we restrict the sample to households expected to be more willing to purchase the plans, estimates of the price elasticity decrease but are still in the same order of magnitude. This is consistent with the literature on the matter. When differentiating the effect according to the HH age, we find that households with heads under the age of 30 are less responsive to changes in price than any other age range. This may be because health insurance risk premiums tend to grow more than proportionally from this age onwards, and households may have less incentive to pay and participate in this market.

Nonetheless, when there is at least one member older than 65 y/o in the household, it responds less to price variations than its counterparts. For example, to a 1% increase in price, a household with at least one member over 65 y/o decreases their demand for VPHI by about 2.8%. Nevertheless, as shown in Table S 4 of the supplemental material, there is no significant difference in behaviour between these two types of households. When analysing the behaviour of households with children under five years of age, the estimates for the price elasticity show that homes with children tend to be more elastic than those without them, possibly responding to parental incentives to take care of the children’s health.

When analysing the results for the income semi-elasticity, the coefficients’ behaviour is similar to those of the premium elasticity when differentiating by the age of the HH. The youngest HH group tends to respond less to changes in their income. Despite continuing to behave as normal goods, the VPHI demand varies only marginally to changes in the income of these households. When there are household members over 65 years of age, an increase in income reflects an increase in VPHI demand compared to households without these members. On the contrary, when there are children under five years of age present in the household composition, the amount of VPHI demanded seems to vary to a lesser extent than when there are none—being all the coefficients of Panel B significant to the values frequently used in the literature. The magnitude of the estimated coefficients is systematically lower than those found in the literature for countries with similar schemes as Colombia. In a study in Spain, authors estimated the income semi-elasticity for a three-period panel data (2008–2014). They report that the cross-sectional models suggest that income effects increase between 2008 and 2014 (0.064 to 0.116, both at 1% significance level). However, when doing the panel estimation for 1928 Spanish households, results tended to decrease and were non-significant at 5% [20]. For Catalonia, one of the most populated regions of Spain, authors report for the whole sample an income elasticity of 1.22 [3]. They also present estimations for age effects, showing that younger individuals have higher income elasticity, except when analysing individuals between 50 and 65 years old. However, this may be related to the quality of the data and the unit of analysis available.

### Possible limitations

This exercise has some limitations to consider. First, the information unit of analysis is the household. This makes it difficult to separate the expense at the individuals’ level and study the base unit of affiliation that is the person. One implication of this limitation is the possible underestimation of the price semi-elasticity since changes in the premium could reduce the number of people affiliated to the service within a household. However, this does not mean that all members participate in a block or stop doing so in the same way. In other words, some household members, perhaps those with the lowest health risk, might decide to stop purchasing insurance. However, others do not, and in this sense, our observation continues to consider the household as a unit spending on VPHI.

A second limitation, perhaps the most important, is given by the quality of the data at the product level. As we have seen, there are a significant number of records with shallow values. This could indicate that the interviewees may have confused payments for other types of insurance or other payments made in health and do not correspond to the premium (i.e., co-payments or moderating fees), despite having a classification by detailed articles (see additional file 2 for the full set of categories). The third is the low percentage of households that report some spending on moderating fees. This implies that market penetration is less than 1%, and even less than 3.5% in middle-income or higher-income households. There are two implications of the underreporting. If it is random, the semi-elasticity would not be affected but the elasticity would be upward biased as the denominator of the expression would be larger. In order to change our conclusion (that the demand is elastic), the overall penetration of VPHI should be of 2% (this is 4.8 times larger than what is calculated in the data). However, the underestimation is likely to be more on middle-income o high income households, who are typically the ones who purchase these products. Our Table 2 shows that in such case the penetration is of 1.49%, and the semi-elasticity is much larger. In this case, the penetration should be twice (3.4%) in order to change our elastic-demand conclusion.

The lack of data on exempt income and deductions for the personal income tax, restricts our ability to compute a premium net of the tax incentives associated to VPHI. Nevertheless, given the size of the tax incentives associated to mortgages, economic dependents, among others, VPHI deductions usually are not part of the effective deductions [26, 27].

An important limitation, which suggest further research in the area, is the impossibility to disentangle the substitution between plans in the system. Our information neither have the names of the policies, nor the coverage of each plan. Therefore, it does not allow us to understand if households change their VPHI on the intensive margin. To the best of our knowledge, Colombia has no more adequate publicly available database to carry out these measurements than the National Household Budget Survey.

## Conclusions

The general estimation of the price-participation elasticity in VPHI for Colombia ranges between -4.67 and -2.32. These estimates were based on a sample of middle to high-income households, who have paid more than 30 USD per month for VPHI. This indicates that a 1% price increase reduces the proportion of households affiliated to a VPHI in the country by about 2.32% to 4.67%, which is higher than those in the literature for other countries. An explanation for this finding can be found in the characteristics of the Colombian health system. Over the last three decades, the health system has reached almost universal coverage with a reasonably broad benefit plan. Added to this is the possibility of accessing health technologies not included in the benefits plan through MIPRES^4^ or judicial mechanisms. In the light of this, households do not have within their top priorities in consumer decisions to acquire voluntary health plans.

These results are of great importance for the design of fiscal policies in Colombia. In the past, authors have concluded that adverse selection is predominant in the Colombian VPHI market [4], implying that subsidies to VPHI coverage can potentially reduce public expenses [4, 22]. A recent study conducted by PROESA confirms that VPHI users substitute public healthcare consumption with private, which translates into savings to the public health system [8]. Hence, in light of a highly elastic VPHI demand, any tax reform aiming to cut VPHI tax benefits is likely to pose a financial burden on the public health system. However, these higher expenses must be contrasted with the fiscal resources that the public health system would have otherwise received. Therefore, the government should estimate the optimal VPHI purchase to reduce any welfare loss that the current arrangement might be generating and introduce the necessary reforms to encourage a more efficient and equitable allocation of resources. Such exercise should also take into consideration the infrastructure investments associated with VPHI, together with risks of increasing fragmentation and inequities in the statutory health care system [10]. 

## Supplementary Information

**Additional file 1.**

**Additional file 2.**

## Data Availability

This project uses secondary publicly available data. All datasets analysed during the current study are available at https://www.dane.gov.co/. Replication code available at https://github.com/androdri1/VPHICOLelasticity. All methods were performed in accordance with the relevant guidelines and regulations.

## Abbreviations

PHI: Private health insurance
VPHI: Voluntary private health insurance
SGSSS: Colombian Social Security Health System (in Spanish: Sistema General de Seguridad Social en Salud)
Fasecolda: Federation of Colombian Insurers (in spanish: Federación de Aseguradores Colombianos)
ENPH: National Household Budget Survey (in Spanish: Encuesta nacional de presupuesto de los hogares)
NHS: National Health Service
Heckit: Heckman Selection Model
AME: Average maginal effect
COP: Colombian Pesos
HH: Head of Household

## Footnotes

1 Other type of out-of-pocket health expenditures are explicitly cover by the questionnaire of the survey. See additional file 2 for further details.

2 For clarity, in additional file 1, readers will be able to find the result of an exhaustive search of prices and coverage of different prepaid medicine plans in the Colombian market, calculated for men of 20 years in Bogotá, Colombia (See Table S1).

3 A complete table with the results of all the steps and models for the Heckit model explain in the empirical strategy section is available in the first Additional file, for the specification sample presented in the main results (see Table S3).

4 MIPRES is a technological tool that allows health professionals to report the prescription of health technologies not financed within the mandatory health scheme or complementary services.
