# Supplementary material for: Price elasticity of demand for voluntary health insurance plans in Colombia
Source: BMC Health Serv Res. 2022 May 9;22:618. doi: 10.1186/s12913-022-07899-2 (PMC9082854; doi:10.1186/s12913-022-07899-2)

**Additional File 1 – Additional Tables and Figures**

**Article:** Price elasticity of demand for voluntary health insurance plans in Colombia

Table S 1. Prepaid Medicine plans prices for Colombia

| Type | Coverage | Premium per month (COP) | Premium per month (USD) |
| --- | --- | --- | --- |
| Prepaid Medicine | Home health services | $ 28.900 | $ 7,64 |
| Prepaid Medicine | Home health services | $ 43.700 | $ 11,56 |
| Prepaid Medicine | Home health services | $ 50.900 | $ 13,46 |
| Prepaid Medicine | Home health services | $ 51.450 | $ 13,61 |
| Prepaid Medicine | Ambulatory health plan | $ 64.550 | $ 17,08 |
| Prepaid Medicine | Dental plan | $ 68.800 | $ 18,20 |
| Prepaid Medicine | Ambulatory health plan | $ 72.100 | $ 19,07 |
| Prepaid Medicine | Dental plan | $ 95.500 | $ 25,26 |
| Prepaid Medicine | Full Coverage | $ 120.000 | $ 31,74 |
| Prepaid Medicine | Full Coverage | $ 121.200 | $ 32,06 |
| Prepaid Medicine | Full Coverage | $ 127.000 | $ 33,59 |
| Prepaid Medicine | Full Coverage | $ 165.707 | $ 43,83 |
| Prepaid Medicine | Full Coverage | $ 176.800 | $ 46,77 |
| Prepaid Medicine | Full Coverage | $ 188.639 | $ 49,90 |
| Prepaid Medicine | Full Coverage | $ 206.759 | $ 54,69 |
| Prepaid Medicine | Full Coverage | $ 223.725 | $ 59,18 |
| Prepaid Medicine | Full Coverage | $ 229.275 | $ 60,65 |
| Prepaid Medicine | Full Coverage | $ 240.000 | $ 63,49 |
| Prepaid Medicine | Full Coverage | $ 251.008 | $ 66,40 |
| Prepaid Medicine | Full Coverage | $ 262.358 | $ 69,40 |
| Prepaid Medicine | Full Coverage | $ 268.200 | $ 70,95 |
| Prepaid Medicine | Full Coverage | $ 270.000 | $ 71,42 |
| Prepaid Medicine | Full Coverage | $ 298.993 | $ 79,09 |
| Prepaid Medicine | Full Coverage | $ 310.400 | $ 82,11 |
| Prepaid Medicine | Full Coverage | $ 312.338 | $ 82,62 |
| Prepaid Medicine | Full Coverage | $ 317.874 | $ 84,09 |
| Prepaid Medicine | Full Coverage | $ 330.400 | $ 87,40 |
| Prepaid Medicine | Full Coverage | $ 339.000 | $ 89,67 |
| Prepaid Medicine | Full Coverage | $ 350.400 | $ 92,69 |
| Prepaid Medicine | Full Coverage | $ 362.901 | $ 96,00 |
| Prepaid Medicine | Full Coverage | $ 363.200 | $ 96,07 |
| Prepaid Medicine | Full Coverage | $ 535.100 | $ 141,55 |
| Prepaid Medicine | Full Coverage | $ 620.600 | $ 164,16 |

Notes: the prices shown in the table are the prices for the price per month for a male individual of 20 years of age in the capital city of Colombia. This information was recovered from: <https://queseguro.co/Comparar/Medicina-Prepagada>, in October 2021. Exchange rate of October 2021 3.780,38 COP/USD.

Table S 2. Marginal effects of household spending on premiums on the probability of participation.

|  | (1) | (2) | (3) | (4) |
| --- | --- | --- | --- | --- |
| Variable | All | Employed | Non-informal | Middle-income or higher |
| **Panel A**: All expenses reported |  |  |  |  |
| Premium semi-elasticity, AME ($p_{i}$) | 0.0213*** | 0.0228*** | 0.0455*** | 0.0771*** |
|  | (0.00323) | (0.00494) | (0.0100) | (0.0168) |
| Income semi-elasticity, AME ($y_{i}$) | -0.00684** | -0.00458 | -0.00938 | -0.0170 |
|  | (0.00298) | (0.00386) | (0.00818) | (0.0135) |
| Observations | 48601 | 31592 | 14384 | 8320 |
| Proportion who spent on VPHI, E[$vphi_{i}$] | 0.00936 | 0.0101 | 0.0211 | 0.0357 |
| Calculated price elasticity ($\varepsilon_{p}$) | 2.274 | 2.259 | 2.151 | 2.160 |
| p-value | 0.000 | 0.000 | 0.000 | 0.000 |
| Calculated income elasticity ($\varepsilon_{y}$) | -0.731 | -0.454 | -0.444 | -0.476 |
| p-value | 0.021 | 0.235 | 0.251 | 0.208 |
| **Panel B**: Expenses over COP 90,000 (30 USD) | |  |  |  |
| Premium semi-elasticity, AME ($p_{i}$) | -0.0194*** | -0.0109*** | -0.0206*** | -0.0346*** |
|  | (0.00352) | (0.00230) | (0.00413) | (0.00715) |
| Income semi-elasticity, AME ($y_{i}$) | 0.0144*** | 0.00921*** | 0.0166*** | 0.0284*** |
|  | (0.00170) | (0.000941) | (0.00170) | (0.00295) |
| Observations | 41153 | 25995 | 14378 | 8320 |
| Proportion who spent on VPHI, E[$vphi_{i}$] | 0.00416 | 0.00485 | 0.00862 | 0.0149 |
| Calculated price elasticity ($\varepsilon_{p}$) | -4.674 | -2.254 | -2.389 | -2.324 |
| p-value | 0.000 | 0.000 | 0.000 | 0.000 |
| Calculated income elasticity ($\varepsilon_{y}$) | 3.459 | 1.901 | 1.921 | 1.905 |
| p-value | 0.000 | 0.000 | 0.000 | 0.000 |
| **Panel C**: Expenses over COP 120,000 (40 USD) | |  |  |  |
| Premium semi-elasticity, AME ($p_{i}$) | -0.0104*** | -0.0147*** | -0.0216*** | -0.0339*** |
|  | (0.00192) | (0.00315) | (0.00450) | (0.00718) |
| Income semi-elasticity, AME ($y_{i}$) | 0.00968*** | 0.0147*** | 0.0207*** | 0.0330*** |
|  | (0.00102) | (0.00159) | (0.00227) | (0.00364) |
| Observations | 41161 | 15148 | 10490 | 6627 |
| Proportion who spent on VPHI, E[$vphi_{i}$] | 0.00360 | 0.00733 | 0.0104 | 0.0164 |
| Calculated price elasticity ($\varepsilon_{p}$) | -2.891 | -2.009 | -2.077 | -2.062 |
| p-value | 0.000 | 0.000 | 0.000 | 0.000 |
| Calculated income elasticity ($\varepsilon_{y}$) | 2.692 | 2.002 | 1.994 | 2.005 |
| p-value | 0.000 | 0.000 | 0.000 | 0.000 |

Notes: Each column presents the marginal effects associated with the parameters $\beta_{1}$ and $\beta_{2}$ of equation (1), corresponding to the semi-elasticity concerning the premium and household income value. The estimation of the simultaneous equations model using data from the ENPH2017 includes (i) estimation of a Heckman selection model (household size is the instrument), (ii) estimation of $\beta_{1}$ and $\beta_{2}$ using the predictions of the premium considering counts the inverse of mills. Each panel corresponds to considering only premium values ​​that exceed a minimum value given that some figures may correspond to some expenses misclassified in the expense article 12530101 "Annual payment of prepaid medicine or complementary health plan." All models include the head of the household, gender dummies, age groups, and educational level. From the household composition, it is considered whether a person over 65 is present and the stratum. There are also region-fixed effects. Standard errors in parentheses. Significance: * 90%, ** 95%, ***99%.

Table S 3. Complete Heckman Selection Model Regressions by steps for the main specification

|  | (1) | (2) | (3) | (4) |
| --- | --- | --- | --- | --- |
| Variable | All | Employed | Non-informal | Middle-income or higher |
| **Panel A:** Probit Results (First Step) |  |  |  |  |
| Household Size | -0.169*** | -0.176*** | -0.191*** | -0.185*** |
|  | (0.035) | (0.041) | (0.043) | (0.042) |
| Log household income | 0.881*** | 0.83*** | 0.85*** | 0.834*** |
|  | (0.063) | (0.072) | (0.075) | (0.074) |
| Male-Head of Household (HH) | -0.079 | -0.096 | -0.087 | -0.085 |
|  | (0.084) | (0.101) | (0.104) | (0.103) |
| HH age: Less than 30 y/o | -0.271 | -0.302 | -0.302 | -0.3 |
|  | (0.26) | (0.268) | (0.274) | (0.272) |
| HH age: 30 to 39 y/o | 0.045 | -0.013 | -0.013 | -0.042 |
|  | (0.115) | (0.127) | (0.129) | (0.13) |
| HH age: 40 to 49 y/o | 0.044 | 0.033 | 0.02 | 0.011 |
|  | (0.108) | (0.117) | (0.12) | (0.12) |
| HH age: 50 y/o or older | . | . | . | . |
|  |  |  |  |  |
|  |  |  |  |  |
| HH Education: Less than high school | -0.457** | -0.629* | -0.428 | -0.538 |
|  | (0.186) | (0.362) | (0.352) | (0.375) |
| HH Education: High school | -0.324* | -0.379 | -0.296 | -0.32 |
|  | (0.168) | (0.266) | (0.275) | (0.273) |
| HH Education: Tertiary education | . | . | . | . |
|  |  |  |  |  |
|  |  |  |  |  |
| At least one member over 65 y/o | 0.227*** | 0.285*** | 0.288*** | 0.292*** |
|  | (0.061) | (0.089) | (0.091) | (0.091) |
| Household income: Low | 2.959 | 2.888 | 2.391 | . |
|  | (98.632) | (123.813) | (91.548) |  |
| Household income: Middle | 3.065 | 3.162 | 2.937 | -0.426*** |
|  | (98.632) | (123.812) | (91.547) | (0.126) |
| Household income: Middle-to-High income | 3.544 | 3.586 | 3.35 | . |
|  | (98.632) | (123.812) | (91.547) |  |
| Region: Atlántico | -0.326** | -0.331** | -0.375** | -0.391** |
|  | (0.13) | (0.154) | (0.162) | (0.162) |
| Region: Bogotá | 0.502*** | 0.496*** | 0.51*** | 0.476*** |
|  | (0.103) | (0.122) | (0.125) | (0.125) |
| Region: New departments | . | . | . | . |
|  |  |  |  |  |
|  |  |  |  |  |
| Region: Eastern | -0.23* | -0.202 | -0.185 | -0.206 |
|  | (0.121) | (0.141) | (0.144) | (0.143) |
| Region: Pacific | -0.144 | -0.153 | -0.141 | -0.156 |
|  | (0.128) | (0.155) | (0.158) | (0.157) |
| HH occupation: Private worker |  | 0.183 | 0.185 | 0.219* |
|  |  | (0.112) | (0.115) | (0.115) |
| HH occupation: Public worker |  | 0.027 | 0.05 | 0.028 |
|  |  | (0.146) | (0.152) | (0.151) |
| HH occupation: Employer |  | 0.079 | 0.072 | 0.097 |
|  |  | (0.175) | (0.178) | (0.177) |
| Constant | -18.564 | -17.877 | -17.912 | -14.341*** |
|  | (98.637) | (123.817) | (91.555) | (1.142) |
| **Panel B:** Regression Results (Second Step) | |  |  |  |
| Log household income | 0.763*** | 0.872*** | 0.837*** | 0.853*** |
|  | (0.255) | (0.267) | (0.255) | (0.258) |
| Male-Head of Household (HH) | -0.154 | -0.291* | -0.288* | -0.275 |
|  | (0.131) | (0.169) | (0.169) | (0.168) |
| HH age: Less than 30 y/o | -0.004 | -0.241 | -0.222 | -0.035 |
|  | (0.458) | (0.477) | (0.478) | (0.479) |
| HH age: 30 to 39 y/o | -0.118 | -0.154 | -0.162 | . |
|  | (0.199) | (0.214) | (0.219) |  |
| HH age: 40 to 49 y/o | . | . | . | 0.177 |
|  |  |  |  | (0.218) |
| HH age: 50 y/o or older | 0.153 | 0.189 | 0.203 | 0.375* |
|  | (0.161) | (0.18) | (0.184) | (0.203) |
| HH Education: Less than high school | -0.415 | -1.569 | -1.486 | -1.536 |
|  | (0.466) | (1.059) | (1.055) | (1.059) |
| HH Education: High school | . | . | . | . |
|  |  |  |  |  |
|  |  |  |  |  |
| HH Education: Tertiary education | -0.363 | -0.026 | -0.116 | -0.083 |
|  | (0.353) | (0.578) | (0.573) | (0.574) |
| At least one member over 65 y/o | -0.014 | 0.203 | 0.188 | 0.2 |
|  | (0.11) | (0.161) | (0.158) | (0.16) |
| Household income: Low | -0.458 | . | . | . |
|  | (0.439) |  |  |  |
| Household income: Middle | . | 0.706 | 0.665 | -0.421 |
|  |  | (0.628) | (0.862) | (0.315) |
| Household income: Middle-to-High income | 0.267 | 1.143* | 1.076 | . |
|  | (0.29) | (0.685) | (0.921) |  |
| Region: Atlántico | -0.036 | -0.2 | -0.213 | -0.223 |
|  | (0.284) | (0.327) | (0.333) | (0.337) |
| Region: Bogotá | 0.292 | 0.72*** | 0.687** | 0.682** |
|  | (0.225) | (0.27) | (0.266) | (0.261) |
| Region: New departments | . | . | . | . |
|  |  |  |  |  |
|  |  |  |  |  |
| Region: Eastern | 0.327 | 0.232 | 0.237 | 0.232 |
|  | (0.209) | (0.253) | (0.252) | (0.253) |
| Region: Pacific | -0.009 | -0.115 | -0.116 | -0.116 |
|  | (0.211) | (0.261) | (0.262) | (0.261) |
| HH occupation: Private worker | . | 0.006 | -0.005 | 0.027 |
|  |  | (0.189) | (0.189) | (0.193) |
| HH occupation: Public worker | . | -0.121 | -0.094 | -0.149 |
|  |  | (0.249) | (0.26) | (0.255) |
| HH occupation: Employer | . | -0.386 | -0.398 | -0.378 |
|  |  | (0.245) | (0.246) | (0.247) |
| Mills Ratio | 0.492 | 0.986** | 0.928** | 0.955** |
|  | (0.388) | (0.443) | (0.415) | (0.426) |
| Constant | -0.493 | -4.341 | -3.486 | -2.956 |
|  | (5.090) | (5.545) | (5.339) | (4.969) |
| **Panel C:** Heckman Selection Probit Results (Third Step) | | |  |  |
| Log premium spending | -2.659*** | -1.299*** | -1.409*** | -1.361*** |
|  | (0.473) | (0.266) | (0.274) | (0.273) |
| Log household income | 1.969*** | 109549*** | 1.133*** | 1.116*** |
|  | (0.217) | (0.093) | (0.098) | (0.097) |
| Male-Head of Household (HH) | -0.404*** | -0.372*** | -0.398*** | -0.368*** |
|  | (0.098) | (0.115) | (0.121) | (0.118) |
| HH age: Less than 30 y/o | -0.386 | -0.527* | -0.563* | -0.524* |
|  | (0.268) | (0.287) | (0.295) | (0.293) |
| HH age: 30 to 39 y/o | -0.706*** | -0.427** | -0.494*** | -0.489*** |
|  | (0.187) | (0.165) | (0.176) | (0.174) |
| HH age: 40 to 49 y/o | -0.401*** | -0.24* | -0.279** | -0.263** |
|  | (0.134) | (0.129) | (0.131) | (0.129) |
| HH age: 50 y/o or older | . | . | . | . |
|  |  |  |  |  |
|  |  |  |  |  |
| HH Education: Less than high school | -0.032 | -1.88*** | -1.825*** | -1.846*** |
|  | (0.204) | (0.475) | (0.428) | (0.487) |
| HH Education: High school | 103717*** | 0.117 | 0.245 | 0.193 |
|  | (0.309) | (0.302) | (0.31) | (0.305) |
| HH Education: Tertiary education | . | . | . | . |
|  |  |  |  |  |
|  |  |  |  |  |
| At least one member over 65 y/o | -0.054 | 0.25*** | 0.248*** | 0.255*** |
|  | (0.074) | (0.09) | (0.092) | (0.092) |
| Household income: Low | -0.11 | 0.87** | -0.463 | . |
|  | (0.251) | (0.418) | (0.445) |  |
| Household income: Middle | 1.063*** | 1.725*** | 0.378 | -0.503*** |
|  | (0.243) | (0.478) | (0.316) | (0.12) |
| Household income: Middle-to-High income | 1.693*** | 2.233*** | 0.887*** | . |
|  | (0.259) | (0.484) | (0.295) |  |
| Region: Atlántico | -0.037 | -0.212 | -0.241 | -0.245 |
|  | (0.137) | (0.151) | (0.159) | (0.16) |
| Region: Bogotá | 0.743*** | 0.912*** | 0.935*** | 0.901*** |
|  | (0.109) | (0.149) | (0.148) | (0.149) |
| Region: New departments | . | . | . | . |
|  |  |  |  |  |
|  |  |  |  |  |
| Region: Eastern | 0.901*** | 0.325* | 0.358* | 0.343* |
|  | (0.238) | (0.184) | (0.183) | (0.186) |
| Region: Pacific | -0.003 | -0.131 | -0.145 | -0.137 |
|  | (0.13) | (0.154) | (0.159) | (0.157) |
| HH occupation: Private worker |  | -0.004 | -0.02 | 0.021 |
|  |  | (0.112) | (0.117) | (0.115) |
| HH occupation: Public worker |  | -0.158 | -0.134 | -0.202 |
|  |  | (0.153) | (0.16) | (0.162) |
| HH occupation: Employer |  | -0.508** | -0.568*** | -0.523** |
|  |  | (0.197) | (0.204) | (0.203) |
| Constant | -0.84 | -4.586** | -2.377 | -1.881 |
|  | (2.626) | (2.24) | (2.715) | (2.578) |
| **Panel D:** Semi-Elasticities and Calculated Elasticities | |  |  |  |
| Premium semi-elasticity, AME ($p_{i}$) | -0.0194*** | -0.0109*** | -0.0206*** | -0.0346*** |
|  | (0.0035) | (0.0023) | (0.0041) | (0.0072) |
| Income semi-elasticity, AME ($y_{i}$) | 0.0144*** | 0.0092*** | 0.0166*** | 0.0284*** |
|  | (0.0017) | (0.0009) | (0.0017) | (0.003) |
| Calculated price elasticity ($\varepsilon_{p}$) | -4.674*** | -2.254*** | -2.389*** | -2.324*** |
|  | (0.8469) | (0.4738) | (0.4791) | (0.4799) |
| Calculated income elasticity ($\varepsilon_{y}$) | 3.459*** | 1.901*** | 1.921*** | 1.905*** |
|  | (0.409) | (0.1942) | (0.1968) | (0.1983) |
| Proportion who spent on VPHI, E[$vphi_{i}$] | 0.00416 | 0.00485 | 0.00862 | 0.0149 |
| Observations | 41,153 | 25,995 | 14,378 | 8,320 |

Notes: Each column presents the results of the specification ran in each of the steps of the Heckman Selection model explain in the methodology section. Panel D presents the marginal effects associated with the parameters and for equation (1), corresponding to the semi-elasticity concerning the premium and household income value. The estimation of the simultaneous equations model using data from the ENPH2017 includes (i) estimation of a Heckman selection model (household size is the instrument), (ii) estimation of and using the predictions of the premium considering counts the inverse of mills. VPHI users are defined as those who reported any expenditure above 90.000 COP (approx 25 USD) in the expense article 12530101, "Annual payment of prepaid medicine or complementary health plan." All models include controls for the head of the household, gender dummies, age groups, and educational level. From the household composition, it is considered whether a person over 65 is present and the income level. There are also region-fixed effects. Standard errors in parentheses. Significance: * 10%, ** 5%, ***1%. Coeficcients marked as “.” were omitted because of collinearity.

Table S 4. Formal tests of equivalence of the calculated elasticity coefficients

|  | Test p-value |
| --- | --- |
| **Panel A**: Log premium spending |  |
| HH Age: |  |
| Age range (1) - (2) | 0.00449 |
| Age range (1) - (3) | 0.0148 |
| Age range (1) - (4) | 0.0000461 |
|  |  |
| At least one member over 65 y/o: |  |
| (No) - (Yes) | 0.504 |
|  |  |
| At least one child under 5 y/o: |  |
| (No) - (Yes) | 0.904 |
| **Panel B**: Log household income |  |
| HH Age: |  |
| Age range (1) - (2) | 0.907 |
| Age range (1) - (3) | 0.729 |
| Age range (1) - (4) | 0.769 |
|  |  |
| At least one member over 65 y/o: |  |
| (No) - (Yes) | 0.378 |
|  |  |
| At least one child under 5 y/o: |  |
| (No) - (Yes) | 0.839 |

*Notes*: The table presents the p-value of tests of equivalence of the calculated elasticity coefficients concerning the value of the premium and household income interacted with the age of the head of the household if there is at least one member of the household over 65 y/o or if there are children under five y/o in the household. The estimation of the simultaneous equations model using data from the ENPH2017 includes (i) estimation of a Heckman selection model (household size is the instrument), (ii) estimation of $\beta_{1}$ and $\beta_{2}$ using the predictions of the premium considering counts the inverse of mills. The model followed is restricted to a minimum premium value of 90,000 COP (approx 25 USD) and for the head of the household to work a non-informal job. All models include the head of the household, gender dummies, age groups, and educational level. From the household composition, it is considered whether a person over 65 is present and the income level. Panels B and D present the p-values for the linear combinations of parameters of the different groups of the interaction term. There are also region-fixed effects. The HH Age ranges are classified as follows (1) Less than 30 y/o, (2) 30 to 39 y/o, (3) 40 to 49 y/o, and (4) 50 y/o or older.

**Figure A.1: Sensibility of the Premium semi-elasticity, AME ($p_{i}$)of demand to the cut-off choice**

*Panel A. Cut-offs from 0 COP to 140.000 COP (46.6 USD)*


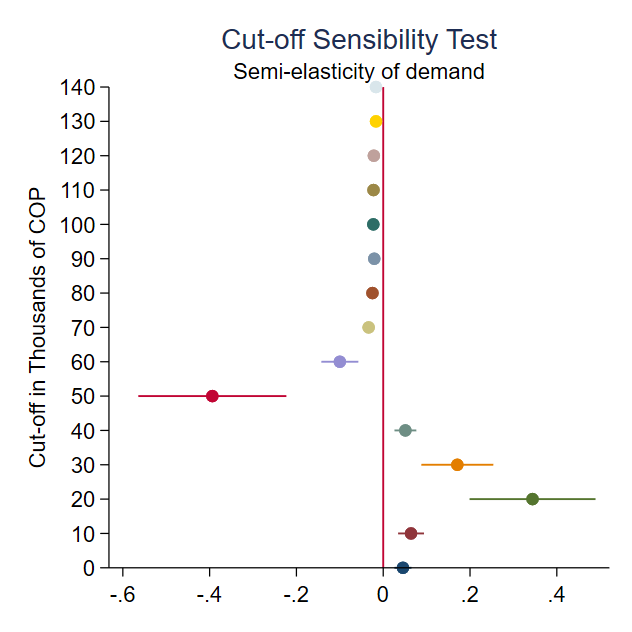


*Panel B. Cut-offs from 60.000 COP (20 USD) to 140.000 COP (46.6 USD)*


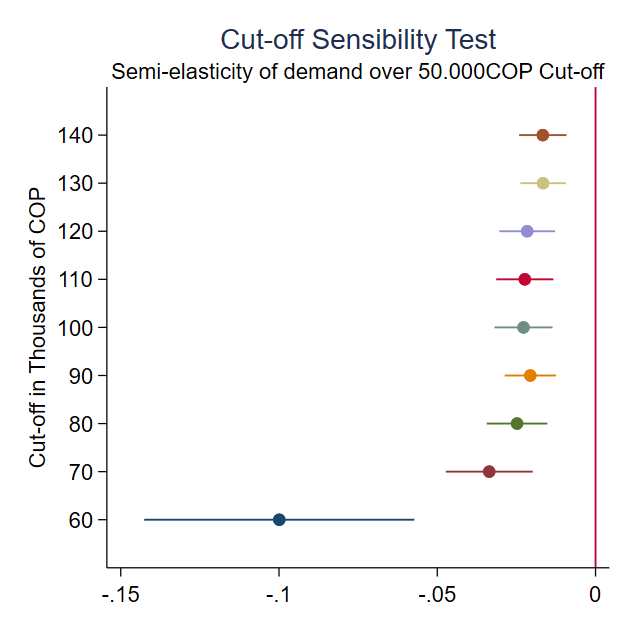

Supplement: Supplementary file 1 — Additional file 1. [file 12913_2022_7899_MOESM1_ESM.docx]
