# Supplementary material for: Price elasticity of demand for voluntary health insurance plans in Colombia
Source: BMC Health Serv Res. 2022 May 9;22:618. doi: 10.1186/s12913-022-07899-2 (PMC9082854; doi:10.1186/s12913-022-07899-2)
Supplement: Supplementary file 2 — Additional file 2. [file 12913_2022_7899_MOESM2_ESM.docx]

**Additional File 2 – Details on the voluntary private health insurance system in Colombia**

VPHI plans are known in Colombia as prepaid medicine or supplemental plans. Ambulance plans are also offered by some companies, but they are not of the interest of this study as they offer very limited coverage in comparison to prepaid medicine or supplemental plans.

All VPHI plans are regulated by the Colombian law ([Ley 1438 de 2011, Capitulo IV](https://www.minsalud.gov.co/Normatividad_Nuevo/LEY%201438%20DE%202011.pdf)) and companies that sell these plans are overseen by the National Superintendent of Health and the Financial Superintendence of Colombia. These plans usually offer supplementary coverage to the mandatory health plan (some offer complementary options as well) (Mossialos & Thomson, 2002). None of them aims to substitute the statutory health care system, as enrolment to the public health system is mandatory for everyone.

Each VPHI plan (a.k.a. prepaid medicine or supplemental plans) and associated premiums are set in accordance with the technical guidelines defined by the National Superintendent of Health ([Circular Externa No. 20 de 2015)](https://normativa.colpensiones.gov.co/colpens/docs/pdf/circular_supersalud_0020_2015.pdf). All VPHI plans and premiums have to be approved by the National Superintendent of Health, before entering the market.

Premiums are set based on age and gender, and they are not allowed to vary with health status. Some plans might offer coverage for pre-existing medical condition (i.e. medical conditions that the person has before buying a VPHI plan), but companies are not forced to cover them according to the Colombian law (Ley 1438 de 2011). For this reason, some companies might ask individuals to undergo a medical exam when signing up the VPHI contract, in order to identify potential pre-existing medical conditions. These conditions, however, cannot change the value of the premium.

VPHI plans can be sold under three different arrangements: (i) individual contracts; (ii) family contracts; and (iii) collective contracts. The last two are also known as group-based VPHI plans. When two individuals or more from the same household decide to purchase a VPHI, they can sign up a family contract. Some companies may offer a 10-15% discount in this case (relative to the price under individual contracts), but it is a fixed discount rate that does not vary with the size of the household. Not all member from the family (household) are necessarily purchasing a VPHI (e.g., some households decide to pay for the parents and the kids). Collective contracts are normally offered to firms that want to offer a VPHI to their employees.

Tax incentives for VPHI are present in Colombia, but they are part of a list of potential personal tax deductions. First, individuals with an annual income below apx 14.000 USD per year (minimum wage would be 3000 USD) do not pay any income tax; this is around 90% of the population in our sample. Second, the taxable income can be reduced by three basic items: (i) having dependents (ex. a child, a non-working spouse) of up to 325 USD, (ii) Interest payment on loans taken out to acquire the taxpayer’s dwelling via mortgage of up to 12,200 USD, (iii) and VPHI of up to 163 USD. On top of this, 25% of the income is exempted for income tax for employees, and for own-employees they can deduct a necessary expenditure to develop their income-generating activity.^[[1]](#footnote-1)^ However, exempt income and deductions cannot exceed 40% of the gross income net of social security taxes. As a result, VPHI deductions usually are at the bottom of the potential tax deductions meaning that they are often not part of the effective deductions.

**Expenditure survey VPHI premium information**

VPHI products are known in Colombia as prepaid medicine, supplemental plans, or private health insurance plans. The information which we use from the survey corresponds to “diversos530101: Annual Premium for Prepaid Medicine and Complementary plans” and it includes premium payments for any of these VPHI products. Other health expenditures are covered in other categories of the expenditure survey. The table below presents all the categories in the survey which are related to out-of-pocket health expenditures. They include expenditure on (full payment, co-payments, co-insurance) medications and other health technologies (including coinsurance for them), healthcare services.

| **Section** | **Variable Name** | **Label** |
| --- | --- | --- |
| g_salud1 |  | Medical Equipment and Products |
|  | g_salud1101 | Dermatological Products |
|  | g_salud1102 | Dermatological Products Sold Only with a Medical Formula |
|  | g_salud1104 | Complete Medical Formula |
|  | g_salud1199 | Other Pharmaceuticals |
|  | g_salud1201 | Medical Supplies |
|  | g_salud1202 | Barrier Contraceptives |
|  | g_salud1301 | Therapeutic Equipment |
| g_salud2 |  | Outpatient Services |
|  | g_salud2101 | General Practitioner Appointment |
|  | g_salud2102 | Specialist Medical Appointment |
|  | g_salud2103 | Orthodontic Specialists Appointment |
|  | g_salud2104 | Bioenergetic Medical Appointment |
|  | g_salud2201 | Dental Services |
|  | g_salud2301 | X-Rays |
|  | g_salud2302 | Laboratory Exams |
|  | g_salud2303 | Auxiliary Medical Services |
|  | g_salud2304 | Rental Therapeutic Equipment |
|  | g_salud2401 | Eps Moderating Fees |
|  | g_salud2402 | Prepaid Medicine Vouchers (Co-Payments) |
| g_salud3 |  | Hospital Services |
|  | g_salud3001 | Inpatient Care |
|  | g_salud3002 | Private Patient Care |
|  | g_salud3003 | Minor Medical Services |
|  | g_salud3004 | Supplemental Hospital Payments |
| g_diversos5301 |  | Medical, Accident and Prepaid Medicine Insurance |
|  | g_diversos530101 | Annual Premium for Prepaid Medicine and Complementary plans* |
|  | g_diversos530103 | Specific Health Insurance (Maternity, Etc.) |

Note: this table was translated from *Cuadernillo* N3, from the questionnaire of the ENPH 2017. * This is the category that we use as a proxy of the VPHI premium.

**Why can household size affect the expenditure of a household on VPHI?**

As described above, premium calculations are always based on the risk profile at the individual level (i.e. the individual as the unit of analysis). The price of group-based VPHI plans, such as plans under family contracts, is also based on the values of individual premiums. Some firms might offer 10-15% discount for plans under family contracts, but the discount rate does not vary with the number of family members under the family contract. This means that the value of the premium (per individual) does not change due to the size of the household, or changes little.

The argument behind using household size variation to explain the decision to buy or not a VPHI is as follows: The larger the size of the household, the higher the total household expenditure of VPHI (see Figure A2.1.). Unlike food and other household goods/services, there are limited economies of scale in the case of VPHI, as described above. As a result, a possibility to keep the share of expenditure on VPHI constant across household sizes (which is what we observe in the data, as shown in Figure A2.2), is to have a decreasing participation in the market according to this variable. Panel A of Table S3 in additional file 1 shows that once we control for other characteristics of a household that could be considered potential predictors of VPHI uptake, such as education, age, type of employer, income level, and region of residence, household size is a strong predictor of VPHI uptake.

Figure A2.1. Distribution of expenditure on VPHI according to the household size

Notes: Expenditures above 90.000 COP (30 USD)

Figure A2.2. Box plots of the expenditure on VPHI's premium as a proportion of HH income, according to household size

Notes: Expenditures above 90.000 COP (30 USD)

1. Extracted from https://home.kpmg/xx/en/home/insights/2021/06/colombia-taxation-of-international-executives.html [↑](#footnote-ref-1)
